# Characterization of *Colletotrichum ocimi* Population Associated with Black Spot of Sweet Basil (*Ocimum basilicum*) in Northern Italy

**DOI:** 10.3390/plants9050654

**Published:** 2020-05-22

**Authors:** Santa Olga Cacciola, Giovanna Gilardi, Roberto Faedda, Leonardo Schena, Antonella Pane, Angelo Garibaldi, Maria Lodovica Gullino

**Affiliations:** 1 Department of Agriculture, Food and Environment, University of Catania, 95123 Catania, Italy; rfaedda@unict.it (R.F.); apane@unict.it (A.P.); 2 Agroinnova—Centre of Competence for the Innovation in the Agro-Environmental Sector, University of Turin, 10095 Turin, Italy; giovanna.gilardi@unito.it (G.G.); angelo.garibaldi@unito.it (A.G.); marialodovica.gullino@unito.it (M.L.G.); 3 Department of Agriculture, Università degli Studi Mediterranea di Reggio Calabria, 89124 Reggio Calabria, Italy; lschena@unirc.it

**Keywords:** *Colletotrichum destructivum* complex, ITS, TUB2, sensitivity to benomyl, pathogenicity

## Abstract

Black spot is a major foliar disease of sweet basil (*Ocimum basilicum*) present in a typical cultivation area of northern Italy, including the Liguria and southern Piedmont regions, where this aromatic herb is an economically important crop. In this study, 15 *Colletotrichum* isolates obtained from sweet basil plants with symptoms of black spot sampled in this area were characterized morphologically and by nuclear DNA analysis using internal transcribed spacers (ITS) and intervening 5.8S nrDNA as well as part of the β-tubulin gene (TUB2) regions as barcode markers. Analysis revealed all but one isolate belonged to the recently described species *C. ocimi* of the *C. destructivum* species complex. Only one isolate was identified as *C. destructivum sensu stricto* (*s.s.*). In pathogenicity tests on sweet basil, both *C. ocimi* and *C. destructivum s.s.* isolates incited typical symptoms of black spot, showing that although *C. ocimi* prevails in this basil production area, it is not the sole causal agent of black spot in northern Italy. While no other hosts of *C. ocimi* are known worldwide, the close related species *C. destructivum* has a broad host range, suggesting a speciation process of *C. ocimi* within this species complex driven by adaptation to the host.

## 1. Introduction

Sweet basil (*Ocimum basilicum* L.), a species of the Labiatae family (tribe Ocimoideae), is an economically important herb crop in several Mediterranean countries and other parts of the world, including France, Israel, Italy, and United States [1]. It is used both as a fresh and dried food spice as well as in traditional medicine. In Italy, approximately 220 ha of sweet basil are grown annually in greenhouses [2] and the leading production area is the Riviera Ligure, Liguria region (northern Italy), where ‘Genovese Gigante’ is the traditional and prevalent cultivar. This cultivar, which accounts for 90% of the total basil-growing area, is at its best when consumed fresh and used for industrial production of the typical pesto sauce [3]. In the last few years, the typical cultivation area of sweet basil has expanded from the Riviera Ligure to the southern provinces of the neighboring Piedmont region. Both in Liguria and Piedmont, basil is mostly grown in a greenhouse, where the drop in temperature during the night and dew are conducive for epidemic outbreaks of fungal diseases such as grey mold, downy mildew, and black spot [1,4]. Black spot is the common name for anthracnose disease of sweet basil and refers to the typical foliar symptoms, either irregular or circular dark-brown spots, which in favorable environmental conditions expand and coalesce into large patches. When lesions desiccate, the leaf lamina shreds. The disease also causes necrotic stem lesions, often resulting in stem girdling and plant death. Black spot is particularly severe in densely planted crops. A similar disease of basil was reported in Florida and the causal agent was identified as *Colletotrichum* sp. [5]. In Italy, an outbreak of black spot of basil was first reported in the Riviera Ligure [6]. A *Colletotrichum* isolate from basil, sampled in this area in 1994 and sent to Centraalbureau voor Schimmelcultures (CBS) for identification, was labeled CBS 298.94 and identified as *Glomerella cingulata*, which was, until recently, regarded as the sexual stage of *C. gloeosporioides*. Consequently, the causal agent of black spot of basil in the Riviera Ligure has long been referred to as *C. gloeosporioides* [4,6]. In the last few years, the taxonomy and nomenclature of the genus *Colletotrichum* has been in continuous flux because of both the vagueness of traditional criteria used to separate the species of this genus, such as the host plant and the morphological characters, and the lack of a comprehensive classification system based on clearly discriminatory genetic markers. Moreover, some species were not adequately typified according to nomenclatural rules. *Colletotrichum gloeosporioides* (e.g., one of the most commonly cited plant pathogens worldwide), was epitypified only about a decade ago [7]. In the last ten years, a comprehensive taxonomic revision of *Colletotrichum* based on molecular and phylogenetic data was provided [8,9,10,11,12,13,14]. These studies have resulted in a much better understanding of phylogenetic relationships and diversity within the genus, and cryptic species have been identified on the basis of multi-gene phylogeny [15,16,17]. Twenty-two species were initially separated within the *C. gloeosporioides* complex, while several other species previously regarded as synonyms of *C. gloeosporioides* were recognized as members of other species complexes [14]. Similarly, multi-locus molecular phylogenetic analysis revealed 31, 18, and 8 separate species within the *C. acutatum*, *C. boninense*, and *C. orbiculare* complexes, respectively [12,13,18]. These studies provided a comprehensive framework based on reliable sequences for all described species where new or undescribed species of *Colletotrichum* could be placed to be classified [19,20,21,22]. Subsequently, for example, additional new species were identified in the *C. gloeosporioides* and *C. boninense* species complexes [23]. In a revision of the *C. destructivum* species complex based on multi-locus DNA sequence analysis [internal transcribed spacers (ITS), glyceraldehyde 3-phosphate dehydrogenase (GAPDH), chitin synthase (CHS-1), imidazoleglycerol-phosphate dehydrogenase (HIS3), actin-like protein (ACT), and β-tubulin (TUB2) gene regions], the isolate CBS 298.94 from basil sourced in the Riviera Ligure was referred to as a new species including only this isolate, distinct from *C. destructivum s.s.* and described formally as *C. ocimi* Damm sp. nov. [24]. *Colletotrichum ocimi* can be identified by its unique ITS, CHS-1, HIS3, ACT, and TUB2 sequences while the GAPDH sequence is the same as that of *C. destructivum s.s.*

The aim of the present study is to better characterize the causal agent of black spot of basil in northern Italy and the species, *C. ocimi*, according with the criterion of defining a novel species on the basis of a collection of isolates [25]. To this end, a larger number of isolates from basil plants showing symptoms of black spot, sourced in the same geographic area where the type isolate of this species originated including Liguria and Piedmont region, were examined.

## 2. Results

### 2.1. Molecular Identification of Isolates

**Figure 1:**
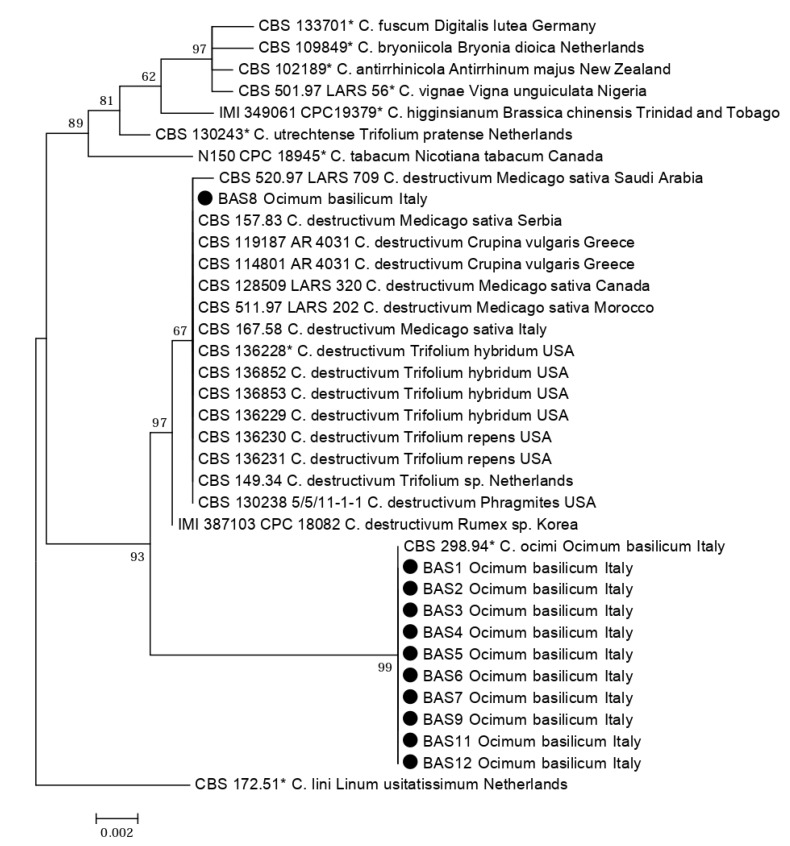
Phylogenetic tree obtained using combined internal transcribed spacers (ITS) and β-tubulin (TUB2) sequences of isolates of *Colletotrichum* spp. collected in the present study from leaves of *Ocimum basilicum* (black dots) along with reference isolates of *C. ocimi*, *C. destructivum*, and other representative species of the *C. destructivum* species complex [24]. An isolate of *C. lini* is used as an outgroup. The evolutionary history was inferred using the maximum likelihood method based on the Tamura–Nei model and the tree with the highest log likelihood is shown. The percentage of trees in which the associated taxa clustered together is shown next to the branches. The asterisks (*) indicate the ex-holotype, ex-epitype or ex-neotype cultures.

The phylogenetic analysis of the combined data set of sequences from ITS and TUB2 regions of all 15 representative *Colletotrichum* isolates collected from basil in northern Italy and included in the present study, along with reference sequences of *Colletotrichum* species separated within the *C. destructivum* complex, produced a phylogenetic tree with a similar topology and high concordance with those reported in previous studies by other authors who revised the systematics of *Colletotrichum acutatum*, *C. boninense*, and *C. destructivum* species complexes using multigene sequence analysis [12,13,24] (Figure 1). All but one isolates from basil were identified as *C. ocimi* because they clustered (bootstrap values 100%) with the ex-type isolate of this species and were clearly distinct from *C. destructivum s*.*s*. Conversely, the sequences of a single isolate (BAS 8) clustered (bootstrap values 100%) with ITS and TUB2 region sequences of reference isolates of *C. destructivum s.s.*, including the ex-epitype CBS136228. Other ITS sequences of isolates sourced from basil identical to those of our isolates of *C. ocimi* were also deposited in GenBank.

### 2.2. Morphological and Cultural Characteristics

**Figure 2:**
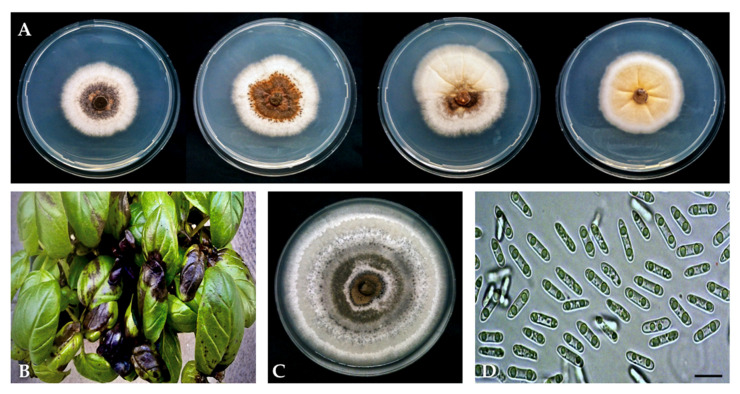
Morphological variability of *Colletotrichum ocimi* colonies: (**A**) From left to right, 13-day-old colonies of isolates BAS 11, BAS 12, BAS 3, and BAS 2, respectively, grown on potato dextrose agar (PDA) at 25 °C in the dark; (**B**) Seedlings of basil ‘Genovese Gigante’ artificially inoculated with *C. ocimi* showing typical symptoms of black spot disease. (**C**) A 13-day-old colony of *C. destructivum* (isolate BAS 8) from basil, grown on PDA at 25° C in the dark. (**D**) Conidia of *C. ocimi* isolate BAS 11 from basil (scale bar = 15 µm).

**Figure 3:**
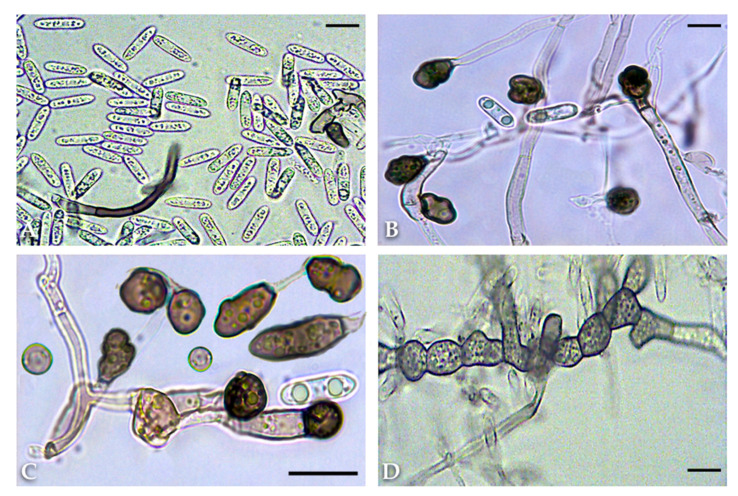
(**A**) Conidia and a curved, septate seta of *Colletotrichum destructivum* (isolate BAS 8 from basil) formed on slide culture. (**B**) Apical and sessile, lobate appressoria formed by isolate BAS 10 of *C. ocimi* on slide culture. (**C**) Various shapes of appressoria formed by isolate BAS 12 of *C. ocimi* on slide culture, ranging from sub-globose and irregular to clavate. (**D**) Appressorium and chlamydospores formed by isolate BAS 8 of *C. destructivum* on slide culture (scale bars = 15 µm).

No *Colletotrichum* isolate from basil grew at 5 and 35 °C, and the optimum growth temperature was 24 °C for all isolates. Plate cultures of all 14 isolates identified as *C. ocimi* on the basis of DNA sequences of the ITS and TUB2 regions were slow-growing, with variable morphology and occasional sectoring (Figure 2A). No significant difference in radial growth rate was observed between isolates of this group (data not shown). The mean radial growth rate (±SD), as determined after nine days of growth on potato dextrose agar (PDA) at 24 °C, was 1.69 ± 0.05 mm day^−1^ (mean of 14 isolates) and ranged from 1.64 to 1.79 mm day^−1^. After six days of growth, colonies were adpressed (flat), without aerial mycelium, sometimes sulcate, white to pale orange, and with either entire or irregular margins; at nine days, colonies of most isolates turned brown with scattered darker conidiomata in the center, whereas colonies of some isolates were orange in the center and did not produce conidiomata (Figure 2A). The reverse of the dishes showed the same colors as the front. No sexual stage was observed. The colonies of the single isolate identified as *C. destructivum* (Figure 2C) grew faster (mean radial growth rate 3 ± 0.1 mm day^−1^); at six days they were adpressed, white in the periphery and gray in the center, with entire margin; at nine days only the external ring of the colony was white while the rest of the colony was gray, with concentric rings of a lighter and darker gray color, sparse darker dots and a central, sticky area of cinnamon color. The reverse of the dishes showed the same colors as the front. No sexual stage was observed. Both species formed hyaline, aseptate, cylindrical, straight or, more rarely, slightly curved conidia with both ends rounded (Figure 2D and Figure 3A). The ranges of dimensions of conidia of the *C. ocimi* isolates overlapped and their average dimensions did not differ significantly (data not shown). The average (±SD) dimensions (length × breadth) of conidia of this group of isolates were 13.07 ± 1.11 (range 8 to 17) × 4.32 ± 0.25 (range 3.5 to 5) µm with an average length/breadth (L/B) ratio of 3.7. The average (±SD) dimensions (length × breadth) of conidia of the *C. destructivum* isolate were 14.9 ± 1.0 (range 14 to 19) × 3.5 ± 0.2 (range 3.5 to 4) µm with an average L/B ratio of 4.4. On slide cultures, isolates of *C. ocimi* formed few setae that measured 40 to 120 µm in length, were brown, smooth-walled, 1–4 septate, straight to curved, with rounded to acute tips.

Conversely, the *C. destructivum* isolate produced numerous setae, directly on hyphae or more often in groups on conidiomata. Setae of this isolate measured 50 to 110 µm in length and were brown, smooth-walled, 1–3-septate, straight to curved, with tips rounded to acute (Figure 3A).

All 14 isolates of *C. ocimi* produced numerous brown to dark brown, smooth-walled appressoria, that formed in an apical or intercalary position on the hyphae or at the apex of germ tubes of conidia. They were single or aggregated in small clusters, frequently sessile, ellipsoidal, clavate, sub-globose or irregular in shape with a lobate to entire margin (Figure 3B,C). Sclerotic intercalary or apical hyphal cells with a melanized wall were sometimes associated with the production of appressoria.

The *C. destructivum* isolate was more reluctant to produce appressoria, while clusters of chlamydospores, cells with brown, melanized, thickened walls, were frequently observed (Figure 3D). Appressoria were single, in an apical position, brown, smooth-walled, clavate, fusiform to ellipsoid, with a lobate, undulate or crenate margin.

### 2.3. Baseline Sensitivity of Isolates to Benomyl

The isolates of diverse *Colletotrichum* species tested were separated into two distinct groups on the basis of their sensitivity to benomyl, highly sensitive isolates with a minimal inhibitory concentration (MIC) <1 µg mL^−1^ and low-sensitive isolates with a MIC >10^2^ μg mL^−1^. The first group comprised all the *C. gloeosporioides s.s.* and *C. karstii* reference isolates, regardless of the host plant and the geographical area of origin, as well as the *C. queenslandicum* and *C. musae* isolates; the second group comprised all the *C. acutatum s.s.*, *C. fioriniae*, *C. godetiae*, and *C. nymphaeae* reference isolates, regardless of the host plant and the geographical area of origin, as well as all 14 *C. ocimi* isolates and the single *C. destructivum* isolate from basil.

### 2.4. Pathogenicity Tests

All isolates from basil, including the *C. destructivum* isolate, induced typical symptoms of black spot on basil seedlings artificially inoculated with a conidial suspension. Seedlings showed minute pinpoint necrotic spots and larger circular or irregular spots on stems and leaves, expanding and coalescing to the entire leaf lamina (Figure 2B), the leaf petiole, and the stem. All leaves of inoculated seedlings showed symptoms and no difference in virulence was observed between fungal isolates. Eventually, seedlings almost completely defoliated and died. Control seedlings did not show symptoms. Results of pathogenicity tests on unwounded leaves using mycelium plugs as inoculum are shown in Table 1.

All five *C. ocimi* isolates and the *C. destructivum* isolate from basil were highly virulent and induced a black, rapidly expanding necrotic halo around the mycelium plug. The same symptoms, but in a less severe form, were observed on leaves inoculated with the *C. fioriniae* isolate from *C. papaya* sourced in the Hawaiian Islands (USA). Both the isolates of *C. godetiae* from olive and the isolate of *C. musae* from banana were weakly pathogenic and induced a very restricted necrotic halo around the mycelium plug. One out of two *C. acutatum* isolates, two out of four *C. gloeosporioides* isolates, and the *C. queenslandicum* isolate produced barely noticeable necrosis. No symptoms were observed on leaves of control seedlings and on leaves inoculated with *C. karstii* or *C. nymphaeae* isolates, the *C. acutatum* isolate OLE or the *C. gloeosporioides* isolates 1765 and C1.

Results of pathogenicity tests on wounded stems are shown in Table 2. All isolates induced necrotic symptoms on stems of inoculated seedlings while symptoms were not observed on control plants. *Colletotrichum ocimi* isolates and the *C. destructivum* isolate from basil were the most virulent among all tested isolates, followed by isolates of *C. fioriniae* and *C. nymphaeae*. Isolates of *C. acutatum* and *C. gloeosporioides* that were non-virulent or barely pathogenic on non-wounded leaves induced quite large lesions on wounded stems. *Colletotrichum karsti* and *C. queenslandicum* isolates were only weakly pathogenic on wounded basil stems. In all experiments, pathogens were re-isolated only from symptomatic, artificially inoculated plants and identified, thus fulfilling Koch’s postulates.

## 3. Discussion

In this study, two closely related *Colletotrichum* species, *C. destructivum s.s.* and *C. ocimi*, were found associated with black spot of basil in Liguria and Piedmont regions. The two species were identified by their ITS and TUB2 sequences and were clearly distinct for some morphological and biological features, such as the colony morphology, the in vitro growth rate, and the ability to produce setae and appressoria. *C. destructivum*, formerly a species complex and quite recently re-defined in a stricter sense, is regarded as a polyphagous pathogen of wild herb plants, forage, and field crops [24]. *C. ocimi*, whose only known host is basil, was separated as a distinct species from *C. destructivum* after the revision of this species complex [24]. *Colletotrichum ocimi* was identified as a causal agent of black spot of basil in Europe and Australia [24,26,27], and it was isolated from basil seeds [26]. In the present study, *C. destructivum* is reported for the first time as a pathogen of basil, although its wide host range includes plants in the family *Lamiaceae* [24,28]. In artificial inoculations, the latter *Colletotrichum* species induced typical symptoms of black spot on basil and was as virulent as *C. ocimi*. However, it was found in a single site in Piedmont. Conversely, *C. ocimi* was by far the dominant species and recovered from basil plants affected by black spot in most sampling sites in both Liguria and Piedmont, confirming its role as a causal agent of this disease in northern Italy. It is fairly common that diverse species of *Colletotrichum* are associated with anthracnose of the same host plant. Multiple *Colletotrichum* species were reported, either singularly or together in mixed infections, on many herbaceous and woody, wild and cultivated plants, including ornamentals and also staple food, industrial, horticultural, and fruit crops [29,30,31,32,33,34,35]. Before the establishment of modern molecular phylogenetic taxonomy, the number of different *Colletotrichum* species associated with a single host plant was underestimated as many sibling species of *Colletotrichum* were referred to as species complex. The diversity of *Colletotrichum* species identified depends on the host plant as well as the number of isolates examined, and the extent of geographical area surveyed. Some species prevail in certain host plants and different geographical areas [36,37]. Moreover, aggressiveness may vary among individual species and isolates within the same species [38,39]. Other local driving factors can modulate the diversity of *Colletotrichum* populations in a crop, including the origin of propagation material, agronomical practices, cultivar susceptibility, and climatic conditions. In the present study, all *Colletotrichum* isolates were obtained from susceptible basil cultivars of the Genovese type, and caused severe losses in field. However, little information related to the susceptibility of the different *Ocimum* species to *C. ocimi* is available. More efforts in developing breeding programs in sweet basil against *C. ocimi* are needed. In total, 10 *Colletotrichum* species associated with avocado anthracnose were identified in Israel using multigene sequence analysis [40], nine were reported to be associated with citrus anthracnose in Europe [23], 13 with olive anthracnose worldwide [41], 11, 15, 12, and 13 with tea, chili pepper, pear, and mango anthracnose, respectively, in China [42,43,44,45], and five with bitter rot of apple in Kentucky [39]. Moreover, the distribution and prevalence of the species may vary in different organs of the plant and according to the season [46,47]. However, not all the *Colletotrichum* occurring together on the same host are pathogenic. The lifestyle of different *Colletotrichum* species, in fact, may vary greatly. Species in this genus may be endophytes, epiphytes, saprobes, opportunistic, hemi-biotrophic or necrotrophic pathogens and may adapt their lifestyle [48]. Thus, when several species occur on the same host, the etiological and epidemiological significance of a single species must be determined both in terms of virulence and prevalence. Both *C. destructivum* and *C. ocimi* have been considered hemi-biotrophic pathogens [23,24,27,28]. In the present study, the results of pathogenicity tests provide further circumstantial evidence of their hemi-biotrophic lifestyle as both species induce severe symptoms on unwounded basil plants. Interestingly, *C. fioriniae*, the only heterologous species which, like *C. destructivum* and *C. ocimi*, is able to induce severe symptoms on unwounded basil plants, was recently reported as a causal agent of leaf anthracnose of oregano (*Origanum vulgare*) in northern Italy [26], suggesting possible cross-infections by this pathogen between plants of the same family. By contrast, all the *Colletotrichum* species tested, although differing in their virulence and regardless of their prevalent lifestyle, infected wounded basil plants. No correlation was found between the results of pathogenicity assays with and without wounding, indicating that results obtained with only artificial wound inoculations to determine the pathogenicity of either novel *Colletotrichum* species or known species on new hosts should be interpreted carefully as the early pathogen–host recognition events are crucial in the infection process by hemi-biotrophic pathogens. The very restricted host range of *C. ocimi*, its prevalence in the field, its high virulence on both wounded and unwounded plants compared to other polyphagous *Colletotrichum* species, and the co-occurrence of a polyphagous, closely related species also pathogenic on basil, such as *C. destructivum*, on the same host are indicative of host-specificity; in addition, it would suggest the hypothesis of a speciation process of *C. ocimi* within the *C. destructivum* species complex driven by adaptation to the host. Ecological evolution processes like this were formerly reported in the *C. acutatum* and *C. gloeosporioides* species complexes. These processes are a consequence of a restricted host range or adaptation to the host and intraspecific differentiation of subpopulations, which in turn may be the result of vegetative compatibility barriers or geographical and reproductive isolation [49,50]. The genetic determinants of host specificity, speciation, and reproductive behaviors of *Colletotrichum* are being studied and species of this genus have been proposed as model systems for understanding the pathogenetic mechanisms of fungi and their strategies to infect host plants [51,52,53,54]. An interesting insight provided by the present study is that *C. destructivum* and *C. ocimi* isolates from basil were inherently not sensitive to benomyl and, in this respect, they grouped with the *C. acutatum* species complex. This benzimidazole fungicide was largely used to control anthracnose diseases of many horticultural crops and ornamentals and in vitro baseline sensitivity to benzimidazole fungicides is a distinctive characteristic separating the *C. acutatum* and *C. gloeosprioides* species complexes [55,56,57,58]. Although, in the case of benomyl, in vitro sensitivity did not necessarily match the actual efficacy in controlling anthracnose in the field, it can be assumed that, in general, differences in sensitivity to fungicides between different *Colletotrichum* species have practical implications for the management of diseases caused by these pathogens, an aspect that deserves to be further investigated by extending the test to other fungicides that are currently in use to control black spot of basil.

## 4. Materials and Methods

### 4.1. Fungal Isolates

A total of 15 *Colletotrichum* isolates from basil, representing a wider population, was characterized in this study. They were obtained between 2007 and 2014 from leaves and cotyledons of basil seedlings of various cultivars with symptoms of black spot in both greenhouse and open field crops in Liguria and the neighboring Piedmont region (Table 3). Each isolate originated from a distinct site and was selected randomly among a variable number of mass isolates (from 40 to 70) obtained by direct isolation from basil plants with typical symptoms of black spot and showing the same colony morphology. All 15 isolates examined in this study were obtained by single-conidium subcultures. Stock cultures were maintained on potato dextrose agar (PDA; Oxoid Ltd., Basingstoke, UK) slants under mineral oil at 5–10 °C.

Isolates of other *Colletotrichum* species and from diverse host-plants were included in pathogenicity and benomyl-sensitivity tests for comparison, as follows: *C. godetiae* (syn. *C. clavatum*) IMI 398854, IMI 398855 and CBS 19332 from *Olea europaea;* and AZJ from *Azalea japonica*; *C. acutatum s.s.* UWS 149, OLE and 67 from *O. europaea*, *Nerium oleander* and *Prunus dulcis*, respectively; *C. fiorinae* ACUVA and 1409 from *Vitis vinifera* and *Carica papaya*, respectively; *C. nymphaeae* 725 and SPL103, both sourced from *Fragaria* × *ananassa* in USA and Italy, respectively; CBS 231.49 from *O. europaea*, *C. gloeosporioides s.s.* A1, 8, 1765, C42, CINA, PEP and C1 from *Annona cherymoia*, *Citrus* × *sinensis, Citrus* sp., *Pistacia vera*, *O. europaea*, *Capsicum annuum,* and *C.* × *limon*, respectively; *C. karstii* C18, C47, CAM and OLF38 from *Citrus × sinensis*, *P. vera*, *Camellia japonica* and *O. europaea*, respectively; *C. queenslandicum* VMIN from *O. europaea* and *C. musae* F15 from *Musa* × *paradisiaca* [37,58].

### 4.2. DNA Extraction, Amplification, and Sequencing

Genomic DNA was extracted from *Colletotrichum* isolates using the method described by Damm et al. [23]. The ITS1–58S–ITS2 region and the fragment of the β-tubulin 2 gene (TUB2) between exons 2 and 6 were amplified and sequenced from a complete panel of isolates as described in a previous paper [59]. Amplified products were analyzed by electrophoresis and single bands of the expected size were purified with the QIAquick PCR Purification Kit (Qiagen, Hilden, Germany) and sequenced with both forward and reverse primers by Macrogen Europe (Amsterdam, The Netherlands). The ChromasPro v. 1.5 software [60] was used to evaluate reliability of sequences and to create consensus sequences. Unreliable sequences in which both forward and reverse sequences, or one or the other, were not successful or contained doubtful bases were re-sequenced. The ITS and TUB2 sequences obtained in the present study were deposited in GenBank with accession numbers MT269781, and from MT269774 to MT269784 (ITS) as well as with accession numbers MT326884 and from MT319098 to MT326883 (TUB2). Validated sequences representative of all species identified within the *C. destructivum* and *C. gloeosporioides* species complexes were phylogenetically analyzed to determine the relationship between different basil isolates and define their taxonomic status in the light of the new molecular criteria for classification [12,13,14,18,24]. Sequences from ex-type or authentic culture were included in the analysis as a reference. Phylogenetic analysis was conducted for the ITS and TUB2 sequences as well as for the combined data set of the two markers using maximum likelihood and Bayesian methods. TOPALi v2 [61] was used to determine the substitution model that best fitted the data. The model HKY + I + G was selected for the Bayesian and maximum likelihood phylogenetic analysis using MrBayes v. 3.1.1 and PhyML v. 2.4.5, respectively, implemented in TOPALi. Bayesian analysis was performed with four runs conducted simultaneously for 500,000 generations with 10% sampling frequency and burn-in of 30%. Maximum likelihood was performed with 100 bootstrap replicates (Table 4, Table S1).

### 4.3. Morphological and Cultural Characteristics

Colony morphology, radial growth rate, and cardinal growth temperature of the isolates from basil were determined on PDA. Colony morphology was examined after 6, 9, 10, and 13 days of incubation at 24 ± 1 °C in the dark. Growth rate was calculated after 9 days incubation. To determine cardinal growth temperatures, 5 mm diameter mycelium plugs taken from the margin of 6-day-old actively growing colonies at 24 ± 1 °C were transferred onto PDA in Petri dishes (9 cm diameter) and incubated at 5, 10, 15, 20, 22, 24, 28, 30, and 35 °C in the dark. Three replicates of each isolate were evaluated, and each experiment was repeated twice. To determine the shape and size of conidia, isolates were inoculated in the center of the Petri dishes (9 cm diameter) containing PDA with a 5 mm diameter mycelial plug and incubated under near-UV light with a 12 h photoperiod for 10 days at 20 °C to enhance sporulation. Conidia were mounted in sterile distilled water (s.d.w.) and observed microscopically at ×1000 magnification. For each isolate, 100 conidia were randomly selected, and length, width, and shape were recorded. Appressoria and setae were produced in slide cultures: small agar squares (about 5 mm) taken from 5-day-old actively growing colonies kept at 24 °C, were placed on sterile glass microscope slides (an agar plug at each extreme). Glasses were incubated in a moist chamber in a sterile Petri dish at 24 °C for 7–10 days in the dark. After gently removing the agar block, microscopic preparations were made by putting a drop of s.d.w. on the slide and adding a cover slip.

### 4.4. Baseline Sensitivity of Isolates to Benomyl

Sensitivity of isolates to benomyl, expressed in terms of MIC, was tested on PDA amended with benomyl (Benlate, Du Pont de Nemours, Wilmington, DE, USA) to obtain final concentrations of 1, 10, and 10^2^ µg per mL of fungicide. Mycelial plugs (5 mm diameter) cut from the margin of 4–5-day-old cultures grown in the dark at 24 °C were plated onto Petri dishes containing approximately 20 mL of amended medium. Dishes were incubated in the dark at 24 ± 1 °C. Growth was recorded after 7 days of incubation. Each isolate was replicated three times per fungicide concentration and was tested twice. Unamended control served as a control.

### 4.5. Pathogenicity Tests

Pathogenicity tests were performed using 5-month-old potted basil seedlings ‘Genovese Gigante’ (8 seedlings per 2 L pot). Seedlings were grown in a sterilized mixture of peat and perlite in a growth chamber, at temperature of 24 ± 2 °C. The growth chamber was lighted by mercury halide and high pressure sodium parabolic aluminic reflector (PAR) lamps of 400 μmol m^−2^ s^−1^ and a photoperiod of 12 h. Three different inoculation methods were used in separate experiments. In the first experiment, only the 15 isolates from basil were tested and inoculum consisted of a conidial suspension (10^6^ conidia mL^−1^) prepared in s.d.w. from 20-day-old cultures grown on PDA at 24 ± 1 °C. Spores were harvested by placing 5 mL of s.d.w. in the plates and gently scraping the mycelium from the agar surface with a long handled glass rod. Finally, spores were counted with an the Burker chamber hemacytometer, and the concentration was adjusted to 10^6^ conidia mL^−1^. The suspension was applied (five pots per fungal isolate) using a manual sprayer to wet the plants thoroughly. The same spore concentration was used for the unwounded pathogenicity tests. Control plants (five pots) were sprayed with s.d.w. After treatment, the pots with seedlings were placed in plastic trays covered with a transparent plastic film to keep a high level of relative humidity (RH) in a growth chamber and kept at 24 ± 1 °C with a 12 h photoperiod. The plastic film was removed three days post-inoculation (dpi). Symptoms were evaluated 10 dpi. This experiment was replicated once and the data of the two experiments were pooled together and analyzed. Data were not shown because all isolates were pathogenic; therefore, only five isolates were chosen as representatives to perform the second trial where the pathogenicity of different *Colletotrichum* species was compared.

In a second series of experiments, five representative *C. ocimi* isolates from basil (BAS 2, BAS 3, BAS 5, BAS 6, and BAS 15), the *C. destructivum* isolate from basil (BAS 8), and representative isolates of other species of *Colletotrichum* were tested, including OLE and 67 (*C. acutatum s.s*.), 1409 (*C. fioriniae*), CINA, 1765, PEP and C1 (*C. gloeosporioides*), IMI 398854 and IMI 398855 (*C. godetiae*), CAM and OLF38 (*C. karstii*), F15 (*C. musae*), SPL 103 (*C. nymphaeae*), and VMIN (*C. queenslandicum*). Expanded leaves (two leaves per seedling) were inoculated on the abaxial surface by placing a PDA agar plug (3 mm diameter) with actively growing mycelium facing down on each side of the midrib, in the center of the lamina. Eight seedlings (a single pot) per fungal isolate were inoculated. Sterile agar plugs were placed on leaves of eight control seedlings. The pots with seedlings were placed in plastic trays covered with a transparent plastic film in a growth chamber and kept at 24 ± 1 °C with a 12 h photoperiod. The film was removed three dpi. The area of necrotic lesion around the plug was measured 14 dpi. The experiment was repeated once and the data of the two experiments were pooled and analyzed together.

In a third series of experiments, the same isolates were tested using a wound-inoculation method. Seedlings (8 in a single 2 L pot per fungal isolate) were wound-inoculated on the stem with a mycelial plug using a wooden toothpick, in accordance with the method described in a previous paper [62]. The length of necrotic lesion was recorded 14 dpi. Eight control seedlings were inoculated with a sterile agar plug. The pots with seedlings were placed in plastic trays covered with a transparent plastic film in a growth chamber and kept at 24 ± 1 °C with a 12 h photoperiod. The film was removed three dpi. This experiment was replicated once and the data of the two experiments were pooled and analyzed together. All fungal isolates included in pathogenicity tests were re-isolated from a representative number of necrotic lesions and identified by morphological characters and sequencing the ITS and β-tubulin regions to confirm their identity.

## 5. Conclusions

This study highlights the need to better understand the biology, ecology, and epidemiology of *C. ocimi* and define its potential host range and actual geographical distribution. Basil is the only known host of this fungus, which has only been reported from Italy and Australia; a report from Florida is not confirmed [63]. The presence of *C. ocimi* in two distant basil cultivation areas may be explained by the spread of this pathogen through contaminated seeds while its restricted distribution might be the consequence of diverse factors, individually or together, such as host specificity, lack of alternative hosts for the inoculum survival and supply of seeds from local producers. Attention should be focused on the genetic and ecological factors determining the virulence and fitness of *C. ocimi*, its widespread occurrence in basil crops of northern Italy, and its ability to adapt specifically to a single host, a trait that differentiates *C. ocimi* from the closely related polyphagous *C. destructivum* and most other *Colletotrichum* species.

## Figures and Tables

**Table 1:** Mean (±SD) area (mm^2^) of necrotic lesions around the mycelium agar plug induced by isolates of various *Colletotrichum* species on artificially inoculated, unwounded basil leaves 14 days post-inoculation (dpi).

*Colletotrichum* Species	Isolate	Mean Area ^a^ (±SD)
*C. acutatum*	OLE	- ^b^
	67	0.26 ± 0.10
*C. destructivum*	BAS 8	82.15 ± 9.25
*C. fioriniae*	1409	7.94 ± 2.46
*C. gloeosporioides*	CINA	0.61 ± 0.29
	1765	- ^b^
	PEP	0.74 ± 0.22
	C1	- ^b^
*C. godetiae*	IMI 398854	1.13 ± 0.35
	IMI 398855	1.22 ± 0.25
*C. karstii*	CAM	- ^b^
	OLF38	- ^b^
*C. musae*	F15	1.79 ± 0.85
*C. nymphaeae*	725	- ^b^
	SPL 103	- ^b^
	CBS 231.49	- ^b^
*C. ocimi*	BAS 2	96.65 ± 14. 42
	BAS 3	85.15 ± 8.25
	BAS 5	88. 96 ± 10.12
	BAS 6	86.45 ± 8.54
	BAS 15	91.75 ± 12.25
*C. queenslandicum*	VMIN	0.74 ± 0.42
Control *^c^*		- ^b^

^a^ Mean of 32 replicates; ^b^ No symptoms; ^c^ Sterile agar plugs.

**Table 2:** Mean (±SD) length (mm) of necrotic lesions on the stem of artificially wound-inoculated basil seedlings induced by various *Colletotrichum* species 14 dpi.

*Colletotrichum* Species	Isolate	Mean Length ^a^ (±SD)
*C. acutatum*	OLE	4.00 ± 0.64
	67	5.40 ± 0.80
*C. destructivum*	BAS 8	9.82 ± 1.25
*C. fioriniae*	1409	7.89 ± 0.92
*C. gloeosporioides*	CINA	6.44 ± 1.03
	1765	5.10 ± 1.50
	PEP	5.10 ± 1.50
	C1	6.15 ± 0.82
*C. godetiae*	IMI 398854	1.89 ± 0.20
	IMI 398855	2.22 ± 0.35
*C. karstii*	CAM	1.11 ± 0.11
	OLF38	1.23 ± 0.15
*C. musae*	F15	1.55 ± 0.17
*C. nymphaeae*	725	7.52 ± 0.81
	SP L103	8.67 ± 0.76
	CBS 231.49	7.48 ± 1.01
*C. ocimi*	BAS 2	13.00 ± 2.03
	BAS 3	11.55 ± 1.08
	BAS 5	10.22 ± 1.05
	BAS 6	9.45 ± 1.55
	BAS 15	12.05 ± 1.45
*C. queenslandicum*	VMIN	3.33 ± 0.23
Control ^b^		- ^c^

^a^ Mean of 16 replicates; ^b^ Sterile agar plugs; ^c^ No symptoms.

**Table 3:** Isolates of *Colletotrichum* sourced from different cultivars of Genovese basil in Liguria and Piedmont (northern Italy) and characterized in this study.

*Colletotrichum* Species	Isolate	Cultivar/Producer	Sourced From	Cropping System	Geographic Origin	Collection Date
*C. ocimi*	BAS 1	Superbo/SAIS	Seedling	Greenhouse ^a^	Grugliasco (TO)	2011
	BAS 2	Gecom FT/SAIS	Seedling	Greenhouse ^a^	Grugliasco (TO)	2011
	BAS 3	Italiko/ANSEME	Seedling	Greenhouse ^a^	Grugliasco (TO)	2011
	BAS 4	Italiano/Olter	Seedling	Greenhouse ^a^	Grugliasco (TO)	2011
	BAS 5	Italiano RCS/Four	Seedling	Greenhouse ^a^	Grugliasco (TO)	2011
	BAS 6	Aromatico/Semencoop	Seedling	Greenhouse ^a^	Grugliasco (TO)	2011
	BAS 7	Profumo/Semencoop	Seedling	Greenhouse ^a^	Grugliasco (TO)	2011
	BAS 9	Italiano classico/SAIS	Leaf	Greenhouse	Albenga (SV)	2007
	BAS 10	Italiko/La Semiorto	Leaf	Open field	Alessandria (AL)	2013
	BAS 11	Italiko/La Semiorto	Leaf	Soilless Greenhouse ^a^	Moncalieri (TO)	2013
	BAS 12	Aromatico Ligure/Semencoop	Leaf	Greenhouse ^a^	Albenga (SV)	2007
	BAS 13	Genovese type ^b^	Leaf	Greenhouse ^a^	Albenga (SV)	2011
	BAS 14	Italiano classico/Pagano	Seedling	Open field	Nichelino (TO)	2014
	BAS 15	Italiano classico/Pagano	Leaf	Open field	Nichelino (TO)	2014
*C. destructivum*	BAS 8	Italiano classico/Pagano	Leaf	Open field	Castagnole Piemonte (TO)	2014

^a^ Experimental greenhouse; ^b^ Unknown cultivar.

**Table 4:** GenBank accession numbers of sequences of the isolates of worldwide origin used as references in phylogenetic analyses.

*Colletotrichum* Species	Isolate	Origin	Host	GenBank Accession		References
				ITS	TUB2	
*C. antirrhinicola*	CBS 102189 *	New Zealand	*Antirrhinum majus*	KM105180	KM105460	[23]
*C. bryoniicola*	CBS 109849 *	Netherlands	*Bryonia dioica*	KM105181	KM105461	[23]
*C. destructivum*	BAS 8	Italy: Piedmont	*Ocimum basilicum*	MT269781	MT326884	This study
*C. destructivum*	CBS 520.97 LARS 709	Saudi Arabia	*Medicago sativa*	KM105217	KM105497	[23]
*C. destructivum*	CBS 157.83	Serbia	*Medicago sativa*	KM105215	KM105495	[23]
*C. destructivum*	CBS 119187, AR 4031	Greece	*Crupina vulgaris*	KM105220	KM105500	[23]
*C. destructivum*	CBS 114801, AR 4031	Greece	*Crupina vulgaris*	KM105219	KM105499	[23]
*C. destructivum*	CBS 128509, LARS 320	Canada	*Medicago sativa*	KM105214	KM105494	[23]
*C. destructivum*	*CBS 511.97, LARS 202*	Morocco	*Medicago sativa*	KM105216	KM105496	[23]
*C. destructivum*	CBS 167.58	Italy	*Medicago sativa*	KM105213	KM105493	[23]
*C. destructivum*	CBS 136228 *	USA	*Trifolium hybridum*	KM105207	KM105487	[23]
*C. destructivum*	CBS 136852	USA	*Trifolium hybridum*	KM105208	KM105488	[23]
*C. destructivum*	CBS 136853	USA	*Trifolium hybridum*	KM105209	KM105489	[23]
*C. destructivum*	CBS 136229	USA	*Trifolium hybridum*	KM105211	KM105491	[23]
*C. destructivum*	CBS 136230	USA	*Trifolium repens*	KM105210	KM105490	[23]
*C. destructivum*	CBS 136231	USA	*Trifolium repens*	KM105212	KM105492	[23]
*C. destructivum*	CBS 149.34	Netherlands	*Trifolium* sp.	JQ005764	JQ005848	[23]
*C. destructivum*	CBS 130238, 5/5/11-1-1	USA	*Phragmites*	KM105218	KM105498	[23]
*C. destructivum*	IMI 387103, CPC 18082	Korea	*Rumex* sp.	KM105221	KM105501	[23]
*C. fuscum*	CBS 133701 *	Germany	*Digitalis lutea*	KM105174	KM105454	[23]
*C. higginsianum*	IMI 349061, CPC 19379 *	Trinidad and Tobago	*Brassica chinensis*	KM105184	KM105464	[23]
*C. lini*	CBS 172.51 *	Netherlands	*Linum usitatissimum*	JQ005765	JQ005849	[23]
*C. ocimi*	BAS 1	Italy: Piedmont	*Ocimum basilicum*	MT269774	MT319098	This study
*C. ocimi*	BAS 2	Italy: Piedmont	*Ocimum basilicum*	MT269775	MT326875	This study
*C. ocimi*	BAS 3	Italy: Piedmont	*Ocimum basilicum*	MT269776	MT326876	This study
*C. ocimi*	BAS 4	Italy: Piedmont	*Ocimum basilicum*	MT269777	MT326877	This study
*C. ocimi*	BAS 5	Italy: Piedmont	*Ocimum basilicum*	MT269778	MT326878	This study
*C. ocimi*	BAS 6	Italy: Piedmont	*Ocimum basilicum*	MT269779	MT326879	This study
*C. ocimi*	BAS 7	Italy: Piedmont	*Ocimum basilicum*	MT269780	MT326880	This study
*C. ocimi*	BAS 9	Italy: Liguria	*Ocimum basilicum*	MT269782	MT326881	This study
*C. ocimi*	BAS 11	Italy: Piedmont	*Ocimum basilicum*	MT269783	MT326882	This study
*C. ocimi*	BAS 12	Italy: Liguria	*Ocimum basilicum*	MT269784	MT326883	This study
*C. ocimi*	CBS 298.94 *	Italy	*Ocimum basilicum*	KM105222	KM105502	[23]
*C. tabacum*	N150, CPC 18945 *	Canada	*Nicotiana tabacum*	KM105204	KM105484	[23]
*C. utrechtense*	CBS 130243 *	Netherlands	*Trifolium pratense*	KM105201	KM105481	[23]
*C. vignae*	CBS 501.97, LARS 56 *	Nigeria	*Vigna unguiculata*	KM105183	KM105463	[23]

* Indicates the ex-holotype, ex-epitype or ex-neotype cultures.

## Supplementary Materials

The following are available online at https://www.mdpi.com/2223-7747/9/5/654/s1, Table S1.
